# Fracture Resistance of Monolithic Zirconia Crowns Depending on Different Marginal Thicknesses

**DOI:** 10.3390/ma15144861

**Published:** 2022-07-12

**Authors:** Seung-Han Kim, Mi-Yeon Yeo, Sun-Young Choi, Eun-Jin Park

**Affiliations:** 1 Department of Prosthodontics, School of Medicine, Ewha Womans University, Seoul 07985, Korea; ihts369@gmail.com (S.-H.K.); annejane1@ewha.ac.kr (S.-Y.C.); 2 Graduate School of Clinical Dentistry, Ewha Womans University, Seoul 07985, Korea; sesy1377@naver.com

**Keywords:** marginal thickness, marginal width, monolithic zirconia, fracture resistance

## Abstract

Under some clinical conditions, the preparation of crowns of limited marginal thickness is inevitable. In such situations, it is questionable whether the same ideal preparation criteria can be applied equally. Since there are only a small number of studies focusing on the fracture resistance with respect to the marginal thickness, there is a need for a study evaluating whether zirconia crowns of limited marginal thickness are clinically acceptable. The purpose of this study is to evaluate the fracture resistance of monolithic zirconia crowns of limited marginal thickness in the posterior area. Methods: Abutments and CAD/CAM zirconia crowns with a marginal thickness of 1.0 mm were set as the control group, while experimental groups A, B, and C possessed reduced marginal thicknesses of 0.8 mm, 0.6 mm, and 0.4 mm, respectively (*n* = 10 per group). Resin-based abutment dies and monolithic zirconia crowns were fabricated using the CAD/CAM technique, and a universal testing machine was used to measure the fracture load value. Fractured specimens were examined with a scanning electron microscope. The data were analyzed using a one-way ANOVA and Bonferroni post hoc test (*p* < 0.05). Results: The means and standard deviations of the fracture load values of the control group and the three experimental groups were as follows: control group (1.0 mm): 3090.91 ± 527.77 N; group A (0.8 mm): 2645.39 ± 329.21 N; group B (0.6 mm): 2256.85 ± 454.15 N; group C (0.4 mm): 1957.8 ± 522.14 N. Conclusions: The crowns fabricated with a CAD/CAM zirconia block with limited marginal thicknesses of 0.6 mm and 0.4 mm showed significantly lower fracture resistance values compared to those with the recommended margin thickness of 1.0 mm.

## 1. Introduction

With patients’ recent increase in interest in esthetics, the use of zirconia, a high-strength ceramic with superior esthetics to dental alloys, is increasing, not only in the anterior region but also in the posterior region [1]. Zirconia is a bioinert ceramic material with mechanical properties close to those of metal, and is receiving great attention as an esthetic prosthetic material in dentistry along with the development of computer-aided design and computer-aided manufacturing (CAD/CAM) technology. It has been reported that monolithic zirconia crowns exhibit fracture resistance that can withstand occlusal loading, even with a limited thickness, when compared to conventional ceramic materials [2,3,4]. By using computerized techniques, the fabrication of monolithic zirconia requires minimal laboratory procedures that use hand instruments. It can also be produced at lower cost and in a shorter manufacturing time, while still achieving greater fracture resistance and a clinically acceptable marginal fit compared to conventional fabrication techniques [5]. When an ideal tooth preparation that meets the necessary standards is achieved, and when the prosthesis is manufactured using standardized zirconia blocks with standardized protocols, the fracture rate is very low. With the increasing use of CAD/CAM zirconia systems, many studies have been conducted on prostheses made of zirconia [6,7]. Sulaiman et al. [8] reported the fracture rate of monolithic zirconia crowns after 7.5 years of clinical service, and the fracture rate of a single monolithic zirconia crown was 0.54%, which was significantly lower than those of layered zirconia crowns and lithium disilicate crowns, which were 2.83% and 1.26%, respectively. Baixauli-López and Mar et al. [9] reported a 98% survival rate for monolithic zirconia crowns in a 5-year prospective study. The authors pointed out that the thickness of the ceramic material might be related to the fracture. Additionally, the fit and accuracy of the margin were evaluated as being some of the important factors affecting the clinical survival rate of a prosthesis [10]. The design and width of the margin vary, including the use of knife edge, chamfer, shoulder, rounded shoulder, and beveled margins, and the amount of reduction also varies depending on the position of the tooth, its morphology, and the material selected for the final prosthesis. In general, in the case of zirconia crowns, 1-mm-deep chamfer or shoulder margins, a 1.0–1.5 mm axial wall reduction, and a 1.5 mm occlusal reduction is recommended in the posterior area [11,12].

However, in clinical situations, there are some cases in which it is difficult to secure a field of view of the posterior area, such as for patients with temporomandibular joint disorder or with a reduced vertical dimension and reduced maximum mouth opening. For such patients, it is difficult to prepare the abutment according to the clinical guidelines, and the amount of preparation on the occlusal or axial surface is limited, resulting in a situation in which the prosthesis is fabricated with a thinner margin than that specified by the manufacturer’s recommendations. According to a study investigating the occlusal convergence, abutment height, and margin design of an abutment for a single crown, an ideal abutment preparation was used in only 4.3% of the total cases. In addition, a clear margin around the entire crown within a single tooth was formed in only 7.5% of cases, and the amount of displacement was higher in the posterior area compared to the anterior region [13]. Other studies investigating the amount of tooth preparation required for posterior crowns in actual clinical practice have confirmed that preparations with thin marginal thicknesses are used in most posterior cases, and the average marginal thickness in the posterior region varies from 0.64 mm to 0.91 mm [14,15,16,17]. Therefore, there is a need for a study to determine whether the same criteria can be applied for clinical situations in which the marginal thickness of the restoration is limited. Previously, many studies have reported on the fracture resistance with respect to the axial taper, abutment height [18,19], margin design [20,21], amount of occlusal reduction, and crown thickness [22], but there are only a small number of studies focusing on the fracture resistance with respect to the marginal thickness. Juntavee et al. [23] reported the fracture strength of monolithic zirconia crowns with respect to the margin thickness, while controlling other variable factors, and compared an 0.8 mm light chamfer margin with a 1.2 mm heavy chamfer margin on a cylinder-shaped die abutment. The results showed a higher fracture load value of 4376 ± 1043 N on the 1.2 mm margin compared to the 0.8 mm margin, which had a value of 3211 ± 778 N. However, as mentioned above, preparations with marginal thicknesses of less than 0.8 mm do occur in clinical situations, and it is questionable whether the same ideal preparation criteria can be applied equally.

Therefore, the purpose of this study is to evaluate the fracture resistance of a zirconia crown with a limited marginal thickness by selecting an experimental group with a marginal thickness of less than 1 mm in a single posterior monolithic zirconia crown manufactured using the CAD/CAM system. The null hypothesis of this study was set as follows: ‘There is no significant difference in fracture resistance according to the thickness of the crown margin’.

## 2. Materials and Methods

### 2.1. Abutment Die Preparation

**Figure 1:**
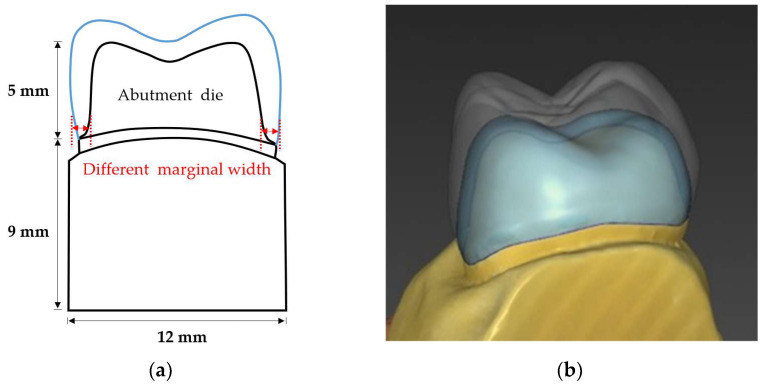
Design of the abutment die and zirconia crown: (**a**) schematic diagram of the abutment die and crown (mesial aspect); (**b**) zirconia crown design.

A prepared maxillary dentiform (CEREC AC Model, Dentsply Sirona, Charlotte, NC, USA) was used for die fabrication. A maxillary left first molar (#26) prepared with a chamfer margin with a width of 1 mm was scanned with an intra-oral scanner (CEREC Primescan, Dentsply Sirona, Charlotte, NC, USA). After obtaining a marginal thickness of 1.0 mm as a control group, experimental groups with margins of 0.8 mm (group A), 0.6 mm (group B) and 0.4 mm (group C) were obtained by using the CEREC design software (Inlab, Dentsply Sirona, Charlotte, NC, USA). The abutment die models were printed with a resin-based printing material (Structomer DentaPro, Structo 3D, MacPherson Rd, Singapore) using a 3D printer (Dentaform, Structo 3D, MacPherson Rd, Singapore). A total of 40 abutment dies (10 for each group) were fabricated in the form of maxillary first molars; the bottoms of the dies were of the same size, measuring 9 mm in height, 12 mm in width, and 18 mm in length, in accordance with the size of the jig being used in the fracture load test. The design of the abutment die and crown is shown in Figure 1, and the mechanical properties of the materials used to fabricate the abutment die are shown in Table 1.

### 2.2. Crown Design

The crowns were designed by a single professional CEREC laboratory technician based on an anatomical crown registered in the design software library. The crown was designed to have an occlusal thickness of 1.5 mm, a buccal/lingual thickness of 1.4 mm, and a cement space of 50 μm for all specimens. All conditions other than the marginal thickness were kept the same.

### 2.3. Crown Fabrication

The zirconia crowns were fabricated using a milling machine (CEREC MC XL, Dentsply Sirona, Charlotte, NC, USA) using a monolithic zirconia block (RAZOR 1100 A3, UNC Int., Seoul, Korea) with parameters as follows: die spacer = 50 μm; proximal contact strength = 25 μm; occlusal contact strength = 25 μm; radial minimum thickness = 50 μm; occlusal minimum thickness adjusted to 1000 μm. The crowns were finished and glazed using a sintering furnace (S-600, Add-in, Gyeonggi-do, Korea) and firing at 1530 °C to room temperature for at least 8 h, in accordance with the manufacturer’s instructions. The sprues were located at least 2 mm apart from the crown margin for marginal accuracy. The marginal thickness of the crown was measured with a metal gauge (4981 M, MEDESY, Maniago, Italy). The chemical composition and mechanical properties of the CAD/CAM zirconia block used in this study are presented in Table 2 and Table 3.

### 2.4. Cementation of Specimens

**Figure 2:**
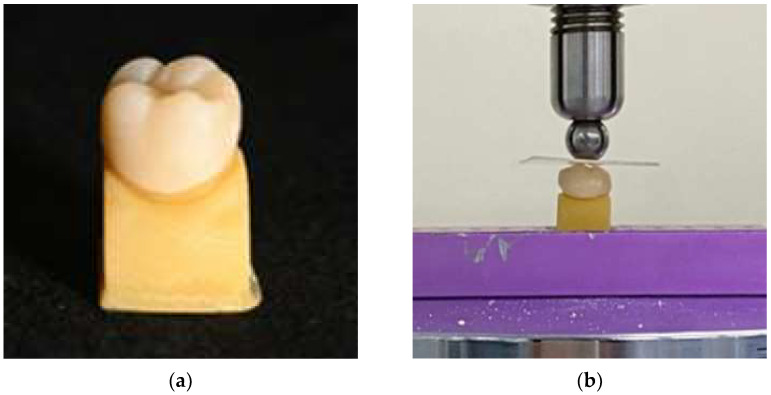
(**a**) CAD/CAM-fabricated zirconia crown and abutment die after cementation. (**b**) Load-to-failure test using a universal testing machine (Instron 3366, Instron Corporation, Norwood, MA, USA) with a crosshead speed of 1.0 mm/min and a 0.64 mm-thick polyethylene sheet (GS025, 3A MEDES, Gyeonggi-do, Korea) placed between the indenter and the specimen.

The crowns were washed and rinsed with ethanol according to the manufacturer’s instructions, and the inner surface of the crown received no additional treatment. Resin-modified glass ionomer cement (Rely X Luting 2, 3M ESPE Dental products, St. Paul, MN, USA) was used to bond the crowns to the abutment dies with finger pressure. Each surface was tack-cured for 5 s, and excessive cement was removed after 2 min in accordance with the manufacturer’s protocol. Bonded die–crown specimens were stored in a water bath (water bath model 9406, Metroden, Seoul, Korea) with distilled water at 37 °C for 24 h before mechanical testing (Figure 2a).

### 2.5. Fracture Load Test

A universal testing machine (Instron 3366, Instron Corporation, Norwood, MA, USA) was used to measure the fracture load values. A spherical stainless-steel indenter with a diameter of 8 mm was placed on the central fossa of the occlusal surface, and a 0.64-mm-thick polyethylene sheet (GS025, 3A MEDES, Gyeonggi-do, Korea) was placed between the indenter and the specimen. A compressive load was applied in the vertical direction to the occlusal surface with a crosshead speed of 1.0 mm/min until fracture, and the fracture load value (N) was recorded (Figure 2b).

### 2.6. Evaluation of Fractured Specimen

After the fracture load test, specimens that showed a load value close to the average were selected and used for microscopic evaluation. The selected specimens were coated and vacuumed for observation. The cross-sections and patterns of the fractured fragments of each experimental group were analyzed using a scanning electron microscope (SEM JEOL-7800F, JEOL Ltd., Tokyo, Japan).

### 2.7. Statistical Analysis

The average and standard deviations of the fracture load values were calculated using the R Project software (R Foundation for Statistical Computing, Vienna, Austria), and one-way ANOVA was performed to evaluate the differences between the experimental groups. As a post hoc test, the Bonferroni test was performed, and the statistical significance was set to 0.05.

## 3. Results

### 3.1. Fracture Load Value

The means and standard deviations of the fracture load values of the control group and the three experimental groups were as follows: control group (1.0 mm): 3090.91 ± 527.77 N; group A (0.8 mm): 2645.39 ± 329.21 N; group B (0.6 mm): 2256.85 ± 454.15 N; group C (0.4 mm): 1957.8 ± 522.14 N. The average and standard deviations of the fracture load values of the control group and the experimental groups are shown in Table 4. It can be seen that the control group showed the highest fracture resistance, and the fracture resistance of the experimental groups decreased with decreasing marginal thickness.

The normality was verified using the Shapiro–Wilk test and a one-way ANOVA test. The Shapiro–Wilk test statistic value was 0.9697 and the *p*-value was 0.3522. Therefore, the fracture resistance values satisfied normality, because the fracture resistance values adopted the null hypothesis of the Shapiro–Wilk test. On the basis of the one-way ANOVA using the fracture load test data, there was a significant difference in fracture resistance depending on the marginal thickness of the monolithic zirconia crown. Bonferroni’s post hoc test was used to determine the mean difference in fracture resistance between the experimental groups on the basis of statistical significance. The results of Bonferroni’s post hoc test indicated that the difference between the mean value of the control group and group A was 445.62 N, which was not statistically significant. However, the mean difference in the value between the control group and group B was 834.06 N, and between the control group and group C it was 1133.11 N, which showed statistical significance. Among the experimental groups, the average difference between group A and group C was 687.59 N, which also showed statistical significance. Therefore, it was confirmed that the control group had higher fracture resistance than group B and group C. Even though group A showed a lower fracture resistance compared to the control group, the result was not statistically significant according to Bonferroni’s post hoc test. The results of the Bonferroni post hoc test are presented in Table 5.

### 3.2. SEM Analysis

**Figure 3:**
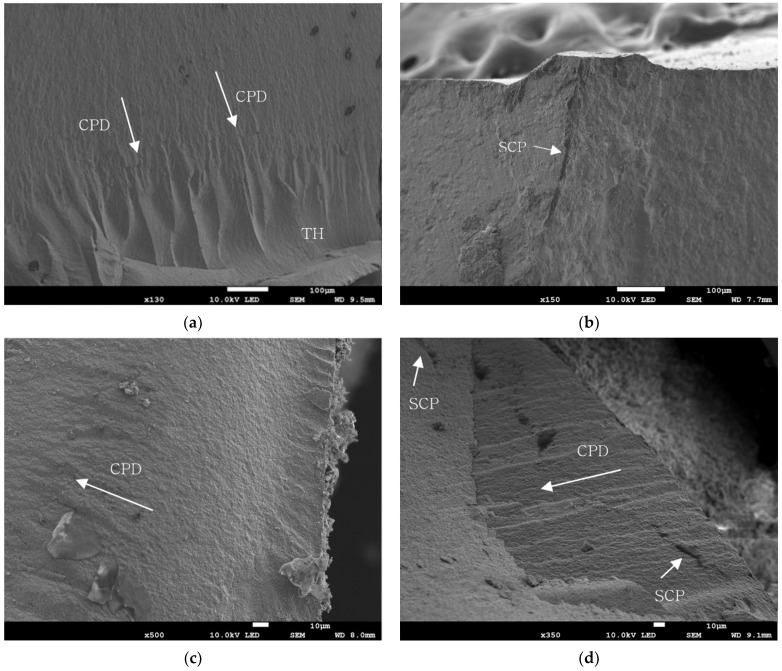
Representative SEM images of the fractured surface of each group: (**a**) fracture image of control group; (**b**) fracture image of group A; (**c**) fracture image of group B; (**d**) fracture image of group C. SCP: stopped crack propagation; CPD: crack propagation direction; TH: twist hackle.

As a result of the fragment analysis, in most of the samples the origin of the fracture was found on the occlusal side, which was in contact with the indenter, while the fragments of the zirconia crown were completely separated, showing a bulk fracture. As a result of the SEM analysis, the fractured specimens showed multiple crack propagations, arrest lines, and twisted hackle lines. The origin of the crack was located on the occlusal surfaces, mostly on the contact point of the loading surface. Multiple stopped crack propagation was observed in groups A and C. Twist hackle lines were peculiarly observed in the control group, and stopped crack propagation lines were distinct in groups A and C. No voids were found at the bonding interface between the abutment die and the crown, indicating that close contact and a proper internal fit had been obtained. The SEM images are shown in Figure 3.

## 4. Discussion

The purpose of this study was to compare the fracture resistance of a monolithic zirconia crown in the posterior region as a function of the marginal thickness. As a result of the experiment performed in this study, it was confirmed that the fracture resistance decreases gradually with the decreasing thickness of the margin. Setting the clinical guideline with a margin thickness of 1 mm as a control group, a decrease in fracture resistance of approximately 300~400 N was observed, with a decrease in the thickness of the margin by 0.2 mm. Although the *p*-value between the control group and group A was 0.23, which showed low statistical significance, the decreases in fracture resistance between the control group and group B and between the control group and group C were statistically significant (*p*-values of 0.0018 and <0.001, respectively). In this study, the maximum load values at the time of crown fracture were compared. The fracture load values of all experimental groups were, on average, 1900 N or higher. All specimens showed higher strength than the average occlusal force of the physiological posterior region reported in the literature, at approximately 700 N [24], with a maximum occlusal force of 1000 N [25].

There have been several studies focusing on the method of fracture load testing of the crown [26,27]. In the present study, as a traditional single-fracture load test, compressive strength was applied to the zirconia crown to measure the load value at fracture. The purpose of the compressive strength test is to measure the resistance of a material to compressive force, and there may be problems such as bending of the specimen during the compression process, resulting in a lower strength being measured than the actual strength. Studies on the fracture resistance of zirconia prostheses have been conducted in various forms, but the experimental results have been inconsistent, with the fracture load values varying among the experiments. Tinschert et al. [28] reported that the fracture resistance of three-unit FPD zirconia was higher than 2000 N, while Sundh et al. [29] reported that the fracture load of zirconia was between 2700 N and 4100 N. The mechanical properties of the abutment die used in the experiment might also affect the fracture load values of monolithic zirconia crowns. In previous studies, the fracture load test protocols for single ceramic restorations have applied various die fabrication methods [30,31,32]. The abutment dies made of natural teeth have an advantage in that they can reproduce a situation similar to the intra-oral condition, but also suffer from the limitation that it is difficult to standardize the specimens between experimental groups. In comparison, metal and resin composite dies have been used as alternatives that can be easily controlled and standardized. Jian et al. [33] reported that the fracture resistance of zirconia crowns cemented to resin dies showed lower fracture load values compared to those of crowns cemented to natural dentin and porous titanium. The resin-based abutment dies used in this study were fabricated using the CAD/CAM technique in order to achieve accurate and equal values for the occlusal reduction and convergence angle, with only the marginal thickness varying. However, the resin-based abutment die, which exhibits a lower elastic modulus than that of natural teeth [34], might be the reason for which the fracture load value of the control group indicating a similar, but lower, fracture resistance compared to previous studies employing similar experimental conditions [23]. Since the condition of the specimens and the measuring method were not standardized in several studies, many studies have made efforts to overcome such limitations. However, despite these efforts, a standardized protocol that is able to reproduce the oral conditions in clinical situations is still lacking. Therefore, there are limitations when predicting the physical properties and clinical prognosis on the basis of a fracture load test alone, since many other factors, including the fracture toughness and flexural strength, are also major factors affecting the durability of a prosthesis [35].

Although the ideal preparation of crowns is important for the long-term prognosis of the restoration, it may be difficult to perform preparations that satisfy all requirements in actual clinical practice. According to a study investigating the occlusal convergence, abutment height, and margin design in abutments for single crowns, an ideal abutment preparation was performed in only 4.3% of the total cases, and the amount of displacement was higher in the posterior area than in the anterior region. In addition, a clear margin around the entire crown within a single tooth was formed only in 7.5% of cases [13]. According to studies investigating the tooth preparations for posterior crowns in actual clinical practice performed by dentists and dental students, it was confirmed that an insufficient marginal thickness was shown in most posterior cases, while the average marginal thicknesses of the posterior region varied from 0.64 mm to 0.91 mm [14,15,16,17]. In the present study, a decrease in fracture resistance was also observed in group A compared to the control group, but there was no statistical significance. Considering the statistically significant differences observed in groups B and C with the control group, and considering that preparations with marginal thicknesses less than 0.8 mm do occur in clinical situations, as mentioned above, it seems that special caution should be applied when the marginal thickness is to be set to 0.8 mm or less.

It is well known that the marginal accuracy is one of the key factors affecting the long-term prognosis of a prosthesis [36]. The same applies to all kinds of restorations, including implant prostheses [37]. In a similar sense, the quality and accuracy of the margin can also influence the fit and accuracy of the provisional restorations, which might consequently effect the prognosis of the final prosthesis. Especially in natural teeth with vitality, the misfit of the provisional restoration can cause pulpal irritation or pulpitis due to bacterial infiltration, which reduces the longevity of the restoration overall [38]. Provisional restorative materials, which show relatively lower fracture resistance compared to the final prosthetic materials, might cause cracks or microfractures while in operation, and the possibility of microleakage will increase with the decreasing thickness of the provisional restorative material.

In this study, the fracture resistance of the monolithic zirconia crowns was measured without considering the loading direction or fatigue. The mechanical test was simply conducted after storing the specimens in 37 °C distilled water for 24 h, without mechanical cyclic loading or thermal cycling, and the loading force was applied only in the vertical direction [39]. By using an abutment die form of a single crown, antagonistic and adjacent teeth conditions were excluded. Therefore, the results of this study only provide limited information on the initial performances of the zirconia crowns under experimental conditions. The results might differ when applied to full-mouth rehabilitation or in implant prostheses in accordance with clinical variations [40].

Thus, in further studies, clinical trials conducted with respect to tooth locations, antagonist and adjacent teeth conditions, and masticatory patterns are needed with a large sample size and long-term assessments.

## 5. Conclusions

The fracture resistance of zirconia crowns with thin marginal thicknesses exhibited lower fracture load values compared to the control group. As expected, the control group exhibited the highest fracture load value of 3090.91 ± 527.77 N. The results showed a gradual decrease in fracture load value with decreasing marginal thickness. Even though the decrease in fracture resistance was not statistically significant up to 0.8 mm, the results showed statistical significance when the marginal width was set below 0.8 mm. Therefore, within the limitations of the study, caution must be taken when employing a thin marginal thickness, especially in clinical situations where the marginal thickness must necessarily be set below 0.8 mm. When preparing abutments for zirconia crowns, an ideal marginal preparation must be used, in accordance with the basic principles. Especially in the posterior area, where the occlusal force is much stronger than in anterior region, caution must be taken when employing thin marginal thicknesses. There are limitations in applying the current findings directly to clinical settings. Thus, in further studies, experimental settings reproducing situations similar to the intra-oral conditions are needed, with larger sample sizes.

## Figures and Tables

**Table 1:** Mechanical properties of the resin die material.

Mechanical Property	Value
Tensile modulus [MPa]	492–583
Maximum tensile strength [MPa]	26–34
Elongation at break [%]	5–6
Flexural modulus [MPa]	173–191
Flexural strength [MPa]	21–33
Shore D hardness	77–85
Viscosity [Pas]	0.7–0.8

**Table 2:** Chemical composition of the zirconia block.

Material	Value [Wt. %]	Test Method
ZrO_2_	88–90	ICP
Y_2_O_3_	7.0–8.0	
SiO_2_	≤0.01	
Fe_2_O_3_	≤0.001	
CaO	≤0.007	
Na_2_O	≤0.004	

**Table 3:** Mechanical properties of the zirconia block.

Mechanical Property	Value	Test Method
Bending strength	1100 ± 50 [Mpa]	3-point-bending ISO 6872
Sintered density	≥6.04 [g/cm^3^]	Archimedes’ method
Transparency	≥46 [%]	Spectrophotometer
Thermal expansion coefficient	10.5 × 10^−6^ [K^−1^]	ASTM test method E 289

**Table 4:** Results of the fracture strength test.

Fracture Strength [N]	Control Group (1.0 mm)	Group A (0.8 mm)	Group B (0.6 mm)	Group C (0.4 mm)
Mean	3090.91	2645.39	2256.85	1957.8
Standard deviation	527.77	329.21	454.15	522.14

**Table 5:** Bonferroni post hoc test.

Groups	Mean Difference [N]	*p*-Value	Lower Control Limit	Upper Control Limit
Control–Group A	445.52	0.2346	−135.386	1026.426
Control–Group B	834.06 *	0.0018	253.154	1414.966
Control–Group C	1133.11 *	<0.001	552.204	1714.016
Group A–Group B	388.54	0.4200	−192.366	969.446
Group A–Group C	687.59 *	0.0130	106.684	1268.496
Group B–Group C	299.05	0.9556	−281.856	879.956

* Statistically significant differences (*p* < 0.05).

## Data Availability

Not applicable.
